# Laying the groundwork to make diversity, equity, and inclusion front and center in clinical and translational research

**DOI:** 10.1017/cts.2023.524

**Published:** 2023-04-24

**Authors:** Leonard E. Egede, Raquel Ruiz, Elise Mosley-Johnson, Sergio A. Aguilar-Gaxiola, Giselle M. Corbie, Consuelo H. Wilkins, Alfred Vitale, L. Ebony Boulware

**Affiliations:** 1 Division of General Internal Medicine, Medical College of Wisconsin, Milwaukee, WI, USA; 2 Center for Advancing Population Science, Medical College of Wisconsin, Milwaukee, WI, USA; 3 Duke University School of Medicine, Durham, NC, USA; 4 University of California, Davis School of Medicine, Sacramento, CA, USA; 5 University of North Carolina Chapel Hill School of Medicine, Chapel Hill, NC, USA; 6 Vanderbilt University Medical Center, Nashville, TN, USA; 7 University of Rochester Clinical and Translational Science Institute, Rochester, NY, USA; 8 Center for Leading Innovation and Collaboration (CLIC), CTSA Program Coordinating Center, Rochester, NY, USA

## Introduction

In the wake of the health and sociopolitical crises following the COVID-19 pandemic, renewed attention has been given to diversity, equity, and inclusion (DEI) work within the clinical and translational science enterprise [1,2]. The COVID-19 pandemic resulted in significant disparities in morbidity and mortality for marginalized and vulnerable populations and coincided with social unrest fueled by persistent, and acute instances of state-sanctioned violence and racial injustice. Researchers and public health workers now find themselves contending with the unsettling truth that can no longer be ignored, that our health care and public health systems are fragmented and woefully inadequate when it comes to mitigating health disparities and ensuring health for all. The clinical and translational science enterprise, along with research centers and academic institutions, need to evaluate their role in these systemic failures. Specifically, failures regarding the lack of progress made in DEI efforts to address the absence of racial/ethnic and gender diversity in institutional leadership, trainees, and staff; as well as limited diversity in clinical trials participation and limited funding for health equity and community-orientated research. Boulware *et al.* in their perspective paper *Combating Structural Inequities – Diversity, Equity, and Inclusion in Clinical and Translational Research* call for a paradigm shift that centers DEI in clinical and translational science work. The piece proposes clear, bold goals and recommended strategies for advancing DEI [2].

The recommended strategies outlined in the perspective paper were developed at the 2020 meeting of the Clinical and Translational Science Awards (CTSA) national consortium meeting. DEI was selected as the primary focus of the 2020 meeting, and goals for discussion included 1) DEI in the field of clinical and translational science, 2) identifying, uncovering, and dismantling sources of systemic racism and bias that contribute to lack of DEI in the field, and 3) to engage in a community-wide dialogue to generate recommendations for bold, sustainable change (2). A committee with DEI expertise was convened and charged with planning the meeting. The planning group utilized the meeting to collect data from attendees on perceived importance and commitment to improving DEI and to guide priority-setting with a pre-meeting survey, post-plenary breakout sessions, and the designation of meeting subgroups that would hold follow-up meetings to synthesize findings and recommendations for dissemination. Of the 796 individuals that registered for the annual meeting, 231 responded to the pre-meeting poll (representing 54 of the 60 CTSA hubs). A total of 479 registrants attended the plenary session, with 98 to 133 participants in each of the 4 post-plenary breakout sessions dedicated to DEI topics. The recommended strategies for achieving DEI in clinical and translational research from the 2020 meeting were captured under 4 broad areas: Leadership, Training, Research, and Clinical Trials.

## Overview of JCTS Theme Issue

As part of dissemination efforts from the meeting, the planning group partnered with the Journal of Clinical and Translational Science to publish a DEI Theme Issue. For this special-themed issue, we set out to highlight advancements in DEI in clinical and translational science. We were particularly interested in developments to promote diverse translational research leaders, develop diverse research trainees, fund, and promote health equity-driven research, and ensuring clinical trials research participants reflect the underlying racial/ethnic diversity of our nation. While these topic areas are not new to those engaged in DEI work, measurable and sustained progress in DEI has eluded the field of clinical and translational science despite its promotion by the NIH and other research organizations for two decades [2]. We grouped our selected manuscripts under 5 topic areas, including Leadership, Training, Research, Clinical Trials, and Community Engagement.

## Theme Issue Highlights

### Leadership

Several manuscripts focus on leadership in clinical and translational science, and they describe efforts to leverage the CTSA program including building upon work started at the 2020 annual meeting to address workforce development and to provide support for more equitable leadership opportunities for underrepresented minorities (URM). This includes critical initiatives which have elevated the CTSA Diversity, Equity, Inclusion, and Accessibility (DEIA) Task Force to a standing committee for the national CTSA consortium [3–5]. Papers also identify crucial roadblocks to achieving DEI goals. For instance, Layne *et al.* highlight how DEI work at the institutional level has historically been expected of Black faculty members, creating an impediment to career advancement and well-being when DEI responsibilities are not recognized as contributing to academic promotion – compounding complex burdens for Black faculty personally affected by systemic racism [6].

### Training

Prioritizing representation in training programs was the highest priority strategy identified for Enhancing DEI in Translational Science Training Programs during the 2020 meeting [7]. Four themes for advancing DEI within clinical and translational science programs aimed at early-stage CTS investigators were also identified and include 1) institutional buy-in; 2) proactive recruitment efforts; 3) an equitable application process; and 4) high-quality, diverse mentorship [8]. In their scoping review on mentoring strategies for junior faculty Williams *et al.* identified very few studies focused on mentoring programs designed to increase diversity, sending a clear signal that existing programs should incorporate purposeful strategies for recruiting diverse mentors and mentees [9]. Rubio *et al.* report women and members of underrepresented groups remain persistently underrepresented among principal investigators in the National Institute of Health’s CTSA program while women have greater representation as participants in the career development and training programs, and non-White individuals are better represented in the training programs [10]. Manuscripts also describe the implementation of novel training curricula addressing structural racism and centering the lived experience of impacted people, as well as training and mentorship programs for URM students, early-career investigators, and clinical research professionals engaged in clinical research recruitment [11–16].

Understanding how well-underrepresented groups are currently represented within the CTSA program will provide critical information toward advancement of DEI goals. The dissemination of the From Insights to Action Resource guide, that provided the framework for the 2020 CTSA meeting, will also guide the National Center for Advancing Translational Sciences (NCATS) goal of workforce diversity and has resulted in gathering important information on the number of diverse graduates still engaged in research [17].

### Health Equity Research

Many manuscripts in this issue focus on identifying ways to diversify participation in clinical research and trials as well as diversifying the biomedical research workforce. The NCATS workgroup paper highlights key areas for leveraging translational science and research to advance DEIA including the use of big data and informatics, prioritizing the health needs of underserved communities, and ensuring evidence-based treatments, and interventions reach the public [18]. Several other papers outline strategies and approaches for improving recruitment – including training and hiring community members to do clinical research, standardizing best practices for community health workers, providing new pathways for clinical research within a medical assistant training program, and providing cultural humility training and supportive environments for clinical research coordinators [19–21]. Using principles of structural competency and structural humility, LeCroy *et al.* propose research interventions should directly address barriers to research participation within and across special populations based on geographic, socioeconomic, and individual constraints by accounting for them within the study design and giving staff more flexibility and resources to accommodate participants [22]. Smith *et al.* remind researchers that while increasing diversity in recruitment efforts is critical to addressing health disparities among affected populations, fair subject selection, and ethical considerations must be applied to ensure fair inclusion, fair burden sharing, and fair opportunity providing historical examples and how the concept was applied to post-sequelae COVID-19 translational studies [23].

Community-based participatory research (CBPR) partnerships are known to be effective for addressing health inequities; however, they are difficult to implement because institutions and funders rarely provide the needed time and resources to build these partnerships. Coombe *et al.* demonstrate that the combination of seed funding and capacity building mentorship can be effective for building strong CBPR partnerships [24]. Interdisciplinary research is essential to addressing ever-increasingly complex scientific problems and is foundational to the functioning of the CTSA hubs. Bengert *et al.* propose using existing institutional electronic databases and grant management systems to evaluate interdisciplinary collaborations, demonstrating the positive impacts of interdisciplinary research on research funding and career advancement [25].

### Clinical Trials

Two manuscripts outline strategies to recruit and enroll diverse study participants and increase racial and ethnic representation of participants, in the context of COVID-19 research. Bell *et al*. present a multicultural and multilingual awareness-raising strategy (26) while the Castellon-Lopez *et al*. demonstrate how a deliberative community engagement approach utilized a Community Consultant Panel comprised of essential workers, community and faith-based organizations, and leaders from racial and ethnic minority communities who helped tailor recruitment materials and inform resources for participants to increase participation in research [27].

Barriers to implementing impactful interventions for chronic disease in real-world settings have resulted in persistent evidence-practice gaps. Nelson *et al.* present findings from a randomized controlled trial intervention using an evidence-based texting self-management support intervention for T2DM within partnering safety clinics and provide recommendations for implementation strategies [28]. To promote clinical trial participation and address mistrust and misinformation surrounding research and clinical trials among underrepresented minority populations, Wolfe *et al*. utilized community health workers (CHWs) as part of a Research Ambassador Program to deliver interactive educational workshop to community members [29].

### Community Engagement

Successful advancements in clinical and translational science are not possible without community engagement in research and implementation programs. Terrance *et al*. describe how Learning Health Systems can prioritize equity by centering diverse community partners’ knowledge and experiences [30]. Stock *et al*. demonstrate how interested racially and ethnically diverse community partners were engaged to lead the creation of a course on structural racism in healthcare and research. To better inform their clinical trial design, Stock *et al.* used virtual community engagement studios with older adults with the goal of better understanding their needs and barriers regarding clinical trial design while soliciting recommendations for improved participation and retention [31]. Sharma *et al.* demonstrate how a community-engaged approach to develop a screening tool by soliciting feedback from community members via virtual Community Advisory Boards and a Community Engagement Studio to address digital inequities [32].

## Conclusion

We have reached a pivotal time in the field of clinical and translational science and research in which we are called to improve DEI and advance health equity through meaningful actions that cultivate diverse leaders and investigators, meaningfully engage our communities, and leverage our capabilities to ensure greater diversity in research. To achieve a vision for diversity and equity in our field, we must embrace a paradigm shift such that DEI and health equity efforts are moved from the fringe to the forefront of institutional missions, culture, and research outcomes. Theme Issue manuscripts provide needed insights on practical strategies to guide next steps, and they also help to lay groundwork for additional work that is needed. While we highlight and applaud growing efforts across the field, we acknowledge that these Theme Issue manuscripts contribute to a groundswell of ongoing efforts which collectively lie at the beginning of an important journey for our field. As the field of clinical and translational science and research looks to the future, there is a need to think differently about how we approach our leadership development and training initiatives and how we engage communities and impacted people not just in research, but within our institutions themselves. We must also explore how research centers, academic institutions, and research funders can be held accountable for making improvements. We hope that this Theme Issue helps to galvanize energy for new thinking across our field to advance and consolidate progress.
